# Pre-referral Rectal Artesunate Treatment by Community-Based Treatment Providers in Ghana, Guinea-Bissau, Tanzania, and Uganda (Study 18): A Cluster-Randomized Trial

**DOI:** 10.1093/cid/ciw631

**Published:** 2016-12-06

**Authors:** Marian Warsame, Margaret Gyapong, Betty Mpeka, Amabelia Rodrigues, Jan Singlovic, Abdel Babiker, Edison Mworozi, Irene Agyepong, Evelyn Ansah, Robert Azairwe, Sidu Biai, Fred Binka, Peter Folb, John Gyapong, Omari Kimbute, Zena Machinda, Andrew Kitua, Tom Lutalo, Melkzedik Majaha, Jao Mamadu, Zakayo Mrango, Max Petzold, Joseph Rujumba, Isabela Ribeiro, Melba Gomes

**Affiliations:** 1 Division of International Health, Karolinska Institutet, Stockholm, Sweden; 2 Dodowa Health Research Centre, Ghana; 3 Malaria Consortium, Kampala, Uganda; 4 Projecto de Saude de Bandim, Guinea-Bissau; 5 Special Programme for Research and Training in Tropical Diseases, World Health Organization, Geneva, Switzerland; 6 Medical Research Council Clinical Trials Unit, London, United Kingdom; 7 Makerere University Medical School, Kampala, Uganda; 8 Greater Accra Regional Health Directorate; 9 Dangme West District Health Directorate, Dodowa, Ghana; 10 National Malaria Control Programme, World Health Organization Uganda Country Office, Kampala; 11 University of Health and Allied Sciences, Ho, Ghana; 12 Medical Research Council, Tygerberg, South Africa; 13 University of Ghana, Accra; 14 National Institute for Medical Research, Dar-es-Salaam; 15 St Augustine University of Tanzania, Mwanza; 16 Preparedness and Response Project, Lugogo House, Kampala, Uganda; 17 Rakai Health Sciences Program, Rakai Project Centre, Entebbe, Uganda; 18 National Institute for Medical Research, Gonja Field Station, Tanzania; 19 Centre for Applied Biostatistics, Sahlgrenska Academy, University of Gothenburg, Sweden; 20 College of Health Sciences, Makerere University, Kampala, Uganda; 21 Drugs for Neglected Diseases, Geneva, Switzerland

**Keywords:** rectal artesunate, coverage, CHWs, mothers, Africa

## Abstract

***Background.*** If malaria patients who cannot be treated orally are several hours from facilities for injections, rectal artesunate prior to hospital referral can prevent death and disability. The goal is to reduce death from malaria by having rectal artesunate treatment available and used. How best to do this remains unknown.

***Methods.*** Villages remote from a health facility were randomized to different community-based treatment providers trained to provide rectal artesunate in Ghana, Guinea-Bissau, Tanzania, and Uganda. Prereferral rectal artesunate treatment was provided in 272 villages: 109 through community-based health workers (CHWs), 112 via trained mothers (MUMs), 25 via trained traditional healers (THs), and 26 through trained community-chosen personnel (COMs); episodes eligible for rectal artesunate were established through regular household surveys of febrile illnesses recording symptoms eligible for prereferral treatment. Differences in treatment coverage with rectal artesunate in children aged <5 years in MUM vs CHW (standard-of-care) villages were assessed using the odds ratio (OR); the predictive probability of treatment was derived from a logistic regression analysis, adjusting for heterogeneity between clusters (villages) using random effects.

***Results.*** Over 19 months, 54 013 children had 102 504 febrile episodes, of which 32% (31 817 episodes) had symptoms eligible for prereferral therapy; 14% (4460) children received treatment. Episodes with altered consciousness, coma, or convulsions constituted 36.6% of all episodes in treated children. The overall OR of treatment between MUM vs CHW villages, adjusting for country, was 1.84 (95% confidence interval [CI], 1.20–2.83; *P* = .005). Adjusting for heterogeneity, this translated into a 1.67 higher average probability of a child being treated in MUM vs CHW villages. Referral compliance was 81% and significantly higher with CHWs vs MUMs: 87% vs 82% (risk ratio [RR], 1.1 [95% CI, 1.0–1.1]; *P* < .0001). There were more deaths in the TH cluster than elsewhere (RR, 2.7 [95% CI, 1.4–5.6]; *P* = .0040).

***Conclusions.*** Prereferral episodes were almost one-third of all febrile episodes. More than one-third of patients treated had convulsions, altered consciousness, or coma. Mothers were effective in treating patients, and achieved higher coverage than other providers. Treatment access was low.

***Clinical Trials Registration.*** ISRCTN58046240.

Prompt, effective malaria treatment prevents death. For severe malaria, artesunate is the treatment of choice. Given injectably, artesunate saves more lives than quinine [1, 2]; given rectally, prior to hospital referral, artesunate prevents both death and disability [3].

Prereferral rectal artesunate adds to an arsenal of inexpensive case management interventions for use in high-mortality settings to reduce illness severity, mortality, and disability. Given appropriately, it reinforces a continuum of care between the home and health facility [4]. It is recommended by the World Health Organization (WHO) [5] and has been added to the community component of Integrated Management of Childhood Illness (IMCI) algorithm which supports early recognition, assessment, treatment, and referral of patients with danger signs in the community [6]. In this algorithm, rectal artesunate treatment is required prior to referral when the child has general danger signs indicative of inability to eat, drink, or suck (*non per os* [NPO]) including: repeated vomiting; recent convulsions; lethargy; or altered/lost consciousness. Facility management after treatment is necessary both to diagnose other infections that might be the cause of life-threatening symptoms and to complete malaria treatment.

The main objective now is to maximize coverage. Treatments only save life if available, accessed, and used. Rectal artesunate takes 4–6 hours to reduce parasitemia. Therefore, the main therapeutic benefit of rectal artesunate is obtained by those who may need several hours to reach a referral facility. During this time the treatment can affect progression of the disease substantially, and reduce the risk of death or permanent disability. Geographical distance from services is a risk factor for death [7]. Even when services are available, logistics and costs anticipated—transport, medications, food, time lost—may prohibit timely access [8–10]. In consequence, many deaths occur without any contact with health services [11]. If used with a rapid diagnostic test, the absolute benefit of treatment increases in relation to the proportion of children with prereferral episodes caused by malaria in the community. The higher the proportion of malaria prereferral episodes, the more important treatment coverage becomes to achieve mortality and disability reduction. Estimates of the burden of prereferral episodes, whether or not caused by malaria, are not generally available because epidemiological data are not collected on prereferral episodes and, by definition, hospital data exclude children who do not reach the facility [12, 13].

Mothers [14, 15] and other community health workers (CHWs) have demonstrated their ability to treat uncomplicated malaria [16, 17], and community-directed treatment programs have been used successfully for treatment access [18]. Traditional birth attendants have been taught to manage pneumonia and neonatal sepsis [19–21], and these and other traditional practitioners are often the first recourse for management of severe disease [22–25]. We anticipated that these types of community-based personnel could be trained to provide rectal artesunate and refer patients for further management, but their performance in increasing access, following up patients, or reinforcing compliance might vary.

We implemented a cluster-randomized study in remote rural villages in Ghana, Guinea-Bissau, Tanzania, and Uganda to obtain evidence on what level of coverage is achievable in practice. Our design enabled us to establish which type of treatment providers were best in reaching patients in need and to provide evidence on the burden of prereferral episodes in children in remote villages. A cluster design responded to the need to measure coverage by community-based treatment providers who are normally responsible for a geographical catchment area.

## METHODS

### Study Area and Population

The intervention was implemented in West-Dangbe district in Ghana; Oio and Biombo districts in Guinea-Bissau; Mubende and Mityana districts in Uganda; and Mtwara district in Tanzania between 2005 and 2008 after assessing treatment-seeking behavior for severe illness [26–28].

### Study Design, Randomization, and Masking

**Figure 1.**
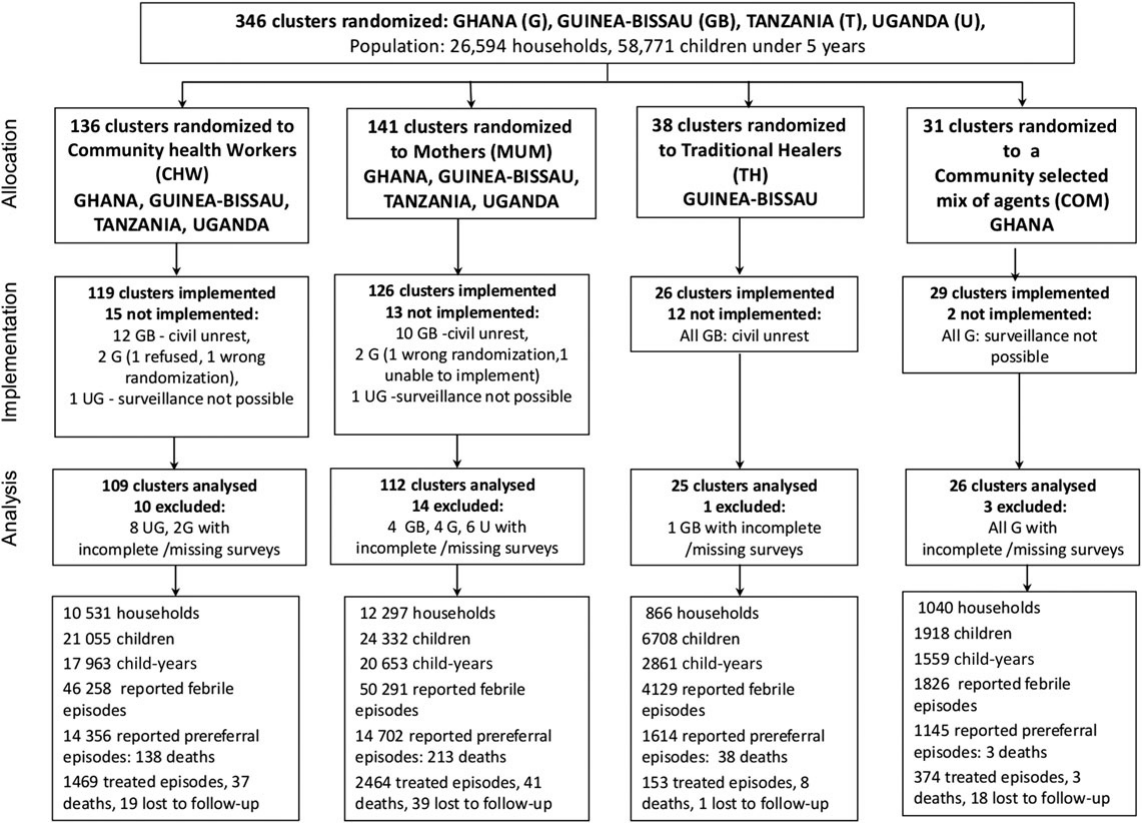
Trial profile of clusters and participants.

Villages without other research interventions, and at least 5 km from the nearest health facility, with at least 400 preschool children and a stable population were eligible for randomization. Each of 346 eligible villages were listed and randomized to different community-based treatment providers (hereinafter called treatment providers) by country, from a computer-generated random allocation sequence (www.randomization.com), concealed until assigned; 42 villages did not implement: civil war (34 villages), inability to conduct surveillance (5 villages), 2 wrong randomizations, and 1 refusal. All remaining villages provided approval for the study. Twenty-eight villages were excluded from the analysis because of incomplete surveys during the implementation period (Figure 1).

All countries chose to compare coverage achieved by CHWs vs mothers (MUM). In Guinea-Bissau and Ghana, an additional treatment provider was chosen: traditional healers (TH) in Guinea-Bissau and a “community chosen” mix of treatment providers (COM) in Ghana. Treatment coverage and relative treatment rates were the primary endpoints, with referral completion rates and adverse outcomes as secondary endpoints (measured individually and by cluster).

Treatment providers knew that their performance would be checked but were not informed that there would be routine surveys of febrile and prereferral episodes (described below) as part of the study or that the number of children they treated was to be monitored. Patients were informed about the drug, its prior history, and where and when to obtain it, but were not aware of allocation (ie, type of cluster). Study personnel were unaware of cluster performance except for verifying data during the period of implementation—defined as the exact period within which treatment and surveillance occurred—from drug provision until drug withdrawal, for each village.

### Coverage Numerator: Provision of Treatment

#### Selection of Treatment Providers

In villages randomized to CHWs, existing CHWs were identified and trained; if not operational, the local procedure for choosing CHWs was applied. Other treatment providers (MUM, TH, COM) were chosen by their communities but had to meet other characteristics (eg, MUMs had to be mothers of young children, THs were practicing traditional healers). All were resident in their village, numerate, generally considered responsible and trustworthy, and willing to provide prereferral treatment at any time. None were paid.

In Uganda it was established, postrandomization, that CHWs were not operational in the study villages despite an official strategy of having CHWs in all villages; thus only in Uganda, selection and training of CHWs and MUMs occurred in parallel. About 3 months after both became operational, a government policy specified that all CHWs should treat suspected malaria with oral prepackaged fixed-combination therapy (sulfadoxine-pyrimethamine + chloroquine; HOMAPAK); this affected 68 villages (31 CHW villages, 37 MUM villages).

#### Training Treatment Providers and Sensitizing Communities

Community posters and pictorial training materials for treatment providers (flipcharts, calendars, case report forms [CRFs]) emphasizing early treatment of suspected severe malaria were developed by a graphic designer. Materials were in the local dialect and emphasized treatment age, signs and symptoms, rectal administration, dose, and the importance of referral to health facilities. Materials were tested for comprehension before use.

Treatment providers in each country were trained together (196 in Ghana, 125 in Guinea-Bissau, 208 in Tanzania, 158 in Uganda) to assess, treat, refer, and follow up patients. The goal was identical training for each treatment provider in a country. Providers who failed certification were retrained or replaced. The number of children who were treated, referred, and followed up was documented. The training curriculum, developed with health authorities, included information on severe malaria, artesunate, symptoms eligible for treatment clinical assessment of patients, informed consent procedures, completion of forms, suppository administration, referral, and follow-up of patients (http://video.who.int/streaming/tdr/WHO-TRA_Malaria_rectal_artesunate_19SEPT2012_en.wmv) [29]. Tanzania had practical sessions on suppository insertion at facilities. Sachets with an artesunate suppository (Abbott artesunate, Scherer capsules, Scanpharm packaging) and coded labels (to be peeled and stuck on the enrollment form) were provided with each dose to each treatment provider together with other materials (stationery, calendars, notebooks).

#### Treatment Eligibility and Referral

Treatment providers assessed patients for eligibility (inability to eat, drink, or suck; repeated convulsions; lethargy/weakness preventing oral medication; repeated vomiting; altered consciousness or coma), obtained individual informed consent from guardians (signed or witnessed and fingerprinted), completed a pictorial treatment form, treated, and referred patients to the nearest health facility. Parasitological examination was not undertaken before treatment [30]. Reinsertion of a fresh suppository was allowed if the first was documented as expelled within 30 minutes. Treated patients were to be followed up within 3–15 days to record outcome and establish compliance with referral advice; patients still unwell at follow-up were to be re-referred again to health facilities. At the referral facility, staff were oriented about the study, advised on reasons for prioritizing children with a referral note, and provided with a facility form to track progress.

### Coverage Denominator: Morbidity and Mortality Surveillance

Episodes needing prereferral artesunate treatment constituted the denominator in calculations of treatment coverage. Approximately every month in each village, all households with children aged <5 years were to be visited (3 attempted interviews) to determine and document whether a child from the household had been febrile since the last visit. Among febrile children, symptoms eligible for rectal artesunate treatment (prereferral episodes) and episode outcomes were recorded. In Ghana and Tanzania, parental actions taken for each prereferral episode were also recorded. Deaths were verified. Surveillance surveys were executed independently by different teams, without knowledge of cluster allocation. Surveys began in each village after the first month of treatment was provided in a village and concluded after drugs were withdrawn.

### Implementation

In each village, implementation began after trained village treatment providers were provided with drugs. Their location and responsibilities in the village for the following 18 months were introduced and approved, usually at a public village meeting; the first village began implementation in 2005. After the first month of correcting CRF completion mistakes, treatment provider performance was supported and observed without interference, except for regular visits when supplies were replenished, drug inventory was scrupulously checked against treatment CRFs, survey registers were collected, and deaths, sequelae, and losses to follow-up were verified. Compliance with referral advice posttreatment was confirmed by a completed facility form and a stamped referral note and patient follow-up. When villages approached their 18th month of operation, visits were made to each village to complete the formalities of study closure, including withdrawing medication, consolidating and verifying drug accountability, and completing final household surveys. No instances of drug misuse were identified in drug reconciliation. Study closure for each village took place over several weeks while inconsistencies in the surveillance and treatment databases were resolved. The trial was monitored.

### Sample Size Calculations

The required sample size was based on anticipated treatment coverage and determined by assuming that the average population of each village would contain about 1600 persons, of whom 20% were expected to be aged <5 years. We anticipated 4 episodes of febrile illness per child during a minimum period of 18 months and (in the absence of data) expected 30% of episodes to be eligible for prereferral treatment*,* so that on average about 384 eligible episodes were predicted to be observed in each village during the study period. The average treatment coverage of CHWs was estimated to be 45% with an intracluster coefficient between villages of 0·25. Under these assumptions, a total of 27 villages per arm would provide 80% power to detect an increase of 10% (from 45% to 55%) in average treatment coverage using a 2-sided *t* test with α = .05. A total of 36 villages per arm would provide 90% power to detect the same difference. We therefore planned to randomize between 27 and 36 villages to each type of treatment provider in each country.

### Statistical Methods

In 3 countries (Ghana, Guinea-Bissau, Uganda), 28 villages (10 CHW, 14 MUM, 1 TH, 3 COM) had incomplete data from household surveys (treated episodes exceeded reported number of episodes needing treatment), and the differences between reported and treated cases could not be reliably reconstructed retrospectively; these villages were excluded from the primary analyses (Figure 1). Contributing villages (109 CHW, 112 MUM, 25 TH, 26 COM) were analyzed by randomized allocation. Treated, nonresident children were excluded from all calculations. Descriptive statistics were used to compare the annualized risks of events (reported fevers, prereferral episodes) during the intervention period per child-year, by cluster and country. The distribution of events were compared by χ^2^ tests with 2-sided *P* values.

### Primary Analysis

The primary objective was to compare the randomized arms in terms of treatment coverage with rectal artesunate in children aged <5 years with symptoms eligible for treatment (NPO). The primary comparison was MUM villages vs CHW (standard-of-care) villages. The coverage difference between clusters was assessed in terms of the odds ratio (OR) and the predictive probability of treatment derived from a logistic regression analysis, adjusting for heterogeneity between clusters (villages) using random effects. The marginal treatment coverage (and its standard error) for each arm was calculated as the average of the predicted probability of treatment for each NPO child (derived from the logistic model). The treatment coverage ratio (ratio of the marginal treatment coverage [for MUM relative to CHW]) and its confidence interval (CI) were derived using the δ method.

We also performed 2 sensitivity analyses for the primary outcome—at the child level and at the cluster level. For the child-level analysis, we used ordinary logistic regression but with robust estimation of standard errors that take clustering by village into account. For the cluster-level analysis, we used a weighted linear regression model of the empirical log-odds of treatment with weights proportional to the inverse of the variance of the log-odds in each village. For each village, the empiric log-odds was calculated as the natural logarithm of the ratio of the number of NPO children treated (a) to the number of those not treated (b) with variance (1/a + 1/b). For villages with a = 0 or b = 0, 0.5 was added to both a and b. All analyses were carried out using Stata software, versions 13 and 14 [31].

## RESULTS

Implementation took place over an average of 19 months. Figure 1 shows the trial organization and the contribution of 24 734 households and 54 013 children <5 years of age to the trial and morbidity, by cluster.

Combining the 4 countries, a total of 102 504 febrile episodes were reported, giving an incidence density rate of about 2.4 febrile episodes per child per year (Table 1). There were comparable risks of febrile episodes by cluster, but not by country, ranging from 3.3 episodes per child-year in Tanzania to 1.1 per child-year in Ghana. In these areas not immediately served by facilities, the prevalence of prereferral episodes averaged 31% (31 817/ 102 504) of all fevers, varying from 26% to 41% among the countries. The average incidence density rate was 0.7 prereferral episodes per child-year exposed, and the highest prevalence of fevers and prereferral episodes was in Tanzania.

**Table 1.** Total Number of Febrile Cases and Prereferral Episodes Reported in Surveillance, and Annualized Risks During the Intervention Period per Child-Year, by Cluster and Country

Type of Episodes	Control		Experimental Treatment Providers						Total	
	Community Health Workers		Mothers		Traditional Healers		Community Mix			
	No.	Risk per Child per Year	No.	Risk per Child per Year	No.	Risk per Child per Year	No.	Risk per Child per Year	No.	Risk per Child per Year
**Febrile episodes (without danger signs)**										
Guinea-Bissau	5743	2.09	3119	1.74	4129	1.44			12 991	1.8
Ghana	1571	1.19	1632	1.07			1826	1.17	5029	1.1
Tanzania	28 047	3.39	30 358	3.28					58 405	3.3
Uganda	10 897	1.94	15 182	1.88					26 079	1.9
Total	46 258	2.58	50 291	2.44	4129	1.44	1826	1.17	102 504	2.4
**Prereferral episodes (with danger signs, NPO)**										
Guinea-Bissau	2315	0.84	1508	0.84	1614	0.56			5437	0.7
Ghana	878	0.67	943	0.62			1145	0.73	2966	0.7
Tanzania	8114	0.98	9675	1.05					17 789	1.0
Uganda	3049	0.54	2576	0.32					5625	0.4
Total	14 356	0.80	14 702	0.71	1614	0.56	1145	0.73	31 817	0.7

Abbreviation: NPO, non per os.

Rectal artesunate treatment was provided to 14% of children with prereferral episodes (4460 treated out of 31 817 reported episodes). Table 2 subdivides treated prereferral episodes by type and by cluster. Because of their higher risk for death, episodes with altered consciousness, coma, or convulsions, hereafter called central nervous system (CNS) episodes, are separated from episodes with other symptoms (weakness, lethargy, repeated vomiting). CNS episodes were 36.6% of all prereferral episodes in children seeking treatment, excluding 39 episodes (0.8%) where accompanying symptoms were not recorded. There were significantly higher episodes with CNS symptoms coming for treatment to CHW vs any other cluster: CHW (41.5%) vs MUM (35.5%): risk ratio (RR), 1.16 (95% CI, 1.07–1.26; *P* < .0003); CHW vs TH (26.8%): RR, 1.54, (95% CI, 1.18–2.02; *P* < .0005); CHW vs COM (28.6%): RR, 1.40 (95% CI, 1.18–1.66; *P* < .0001).

**Table 2.** Episodes Treated With Prereferral Rectal Artesunate, All Countries

Type of Episodes	Control	Experimental Treatment Providers			Total
	Community Health Workers	Mothers	Traditional Healers	Community Mix	
Treated episodes, by symptom^a^					
Without CNS symptoms^a^	855 (58.2)	1569 (63.7)	111 (72.6)	253 (67.7)	2788 (62.5)
CNS symptoms^a^	609 (41.5)	876 (35.5)	41 (26.8)	107 (28.6)	1633 (36.6)
Not documented	5 (0.3)	19 (0.8)	1 (0.6)	14 (3.7)	39 (0.9)
Compliance with referral advice					
Went to facility	1259 (85.7)	1982 (80.4)	97 (63.4)	294 (78.6)	3632 (81.4)
No compliance	192 (13.1)	443 (18.0)	53 (34.6)	76 (20.3)	764 (17.2)
Used alternative care	18 (1.2)	39 (1.6)	3 (2.0)	4 (1.1)	64 (1.4)
Deaths among treated episodes					
Alive	1413 (96.2)	2384 (96.8)	144 (94.1)	353 (94.4)	4294 (96.3)
Died	37 (2.5)	41 (1.6)	8 (5.2)	3 (0.8)	89 (2.0)
Lost to follow-up	19 (1.3)	39 (1.6)	1 (0.7)	18 (4.8)	77 (1.7)
Total, No.	1469	2464	153	374	4460

Data are presented as No. (%).

Abbreviation: CNS, central nervous system.

^a^ CNS symptoms are defined as altered consciousness, coma, or convulsions.

Completion of referral advice at a facility was documented at follow-up within a median of 6 days for 98.6% of patients. Overall, 81.4% of patients proceeded to facilities for further care but referral completion was better with CHWs vs MUMs: 85.7% vs 80.4% (RR, 1.06 [95% CI, 1.03–1.09]; *P* < .0001) or CHW and MUM combined vs COM and TH villages combined: 83.6% vs 75.2% (RR, 1.11 [95% CI, 1.06–1.17]; *P* < .0001). The death rate was higher in the TH cluster than elsewhere (RR, 2.7 [95% CI, 1.35–5.58]; *P* = 0040).

The primary objective was to predict the likelihood of a child being treated by type of treatment provider. Results are given in Table 3, adjusting for heterogeneity between villages by a random effect for cluster in logistic regression analysis. The results vary by country, with no difference in the predicted probability of a child being treated in a MUM vs CHW cluster in Guinea-Bissau or Uganda but double the probability of being treated in Tanzania or Ghana. The overall ORs and CIs reflect these results: an OR of 1.84 (95% CI, 1.20–2.83; *P* = .005) translates into a 1.67 higher overall predicted probability of a child being treated in MUM vs CHW clusters.

**Table 3.** Likelihood of Being Treated, Community Health Workers Versus Mother Cluster: Logistic Regression Results, Adjusting for Heterogeneity Between Villages by a Random Effect for Cluster

Country & Cluster	Odds of Being Treated	Predictive Probability of Treatment
	Odds Ratio (95% CI); *P* Value	Ratio (95% CI); *P* Value
Guinea-Bissau		
MUM	0.06 (.02–.19)	0.06 (.02–.17)
CHW	0.05 (.02–.15)	0.05 (.02–.14)
Ratio	1.17 (.23–5.85); .8523	1.16 (.25–5.35); .8522
Ghana		
MUM	0.73 (.39–1.36)	0.42 (.29–.60)
CHW	0.27 (.14–.52)	0.22 (.13–.36)
Ratio	2.66 (1.09–6.52); .0321	1.96 (1.06–3.65); .0333
Tanzania		
MUM	0.17 (.12–.24)	0.15 (.11–.20)
CHW	0.08 (.06–.11)	0.07 (.05–.10)
Ratio	2.19 (1.34–3.57); .0017	2.02 (1.30–3.12); .0017
Uganda		
MUM	0.32 (.18–.55)	0.24 (.16–.37)
CHW	0.23 (.13–.42)	0.19 (.12–.31)
Ratio	1.37 (.60–3.10); .4551	1.28 (.67–2.44); .4559
**Overall, adjusted for country**		
MUM	0.22 (.16–.29)	0.17 (.13–.22)
CHW	0.12 (.09–.16)	0.10 (.08–.13)
**Ratio**	**1.84 (1.20–2.83); .0050**	**1.67 (1.17–2.38); .0051**

Abbreviations: CHW, community health worker; CI, confidence interval; MUM, mother.

**Figure 2.**
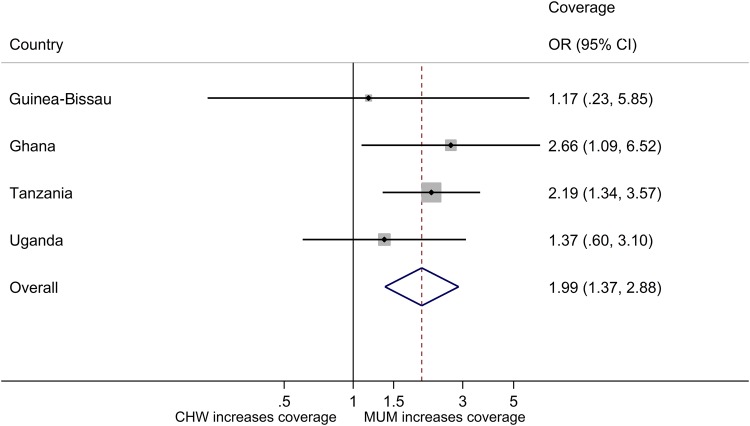
Coverage: odds ratios (ORs) and 95% confidence intervals (CIs) for treatment, mother (MUM) vs community health worker (CHW) clusters.

**Figure 3.**
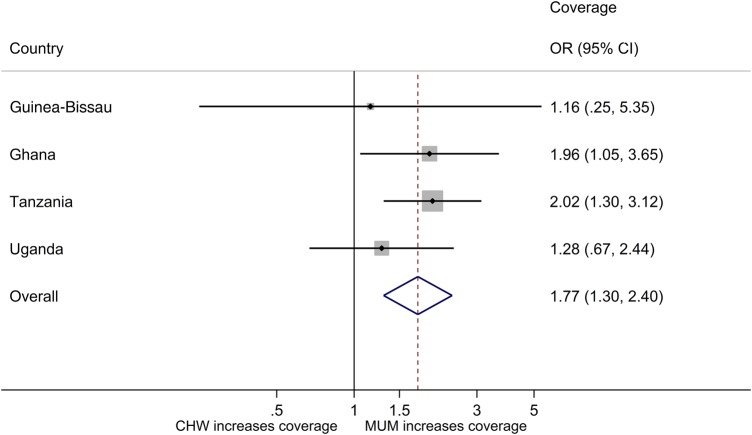
Coverage: adjusted predicted probability ratio of treatment (odds ratios [ORs] and confidence intervals [CIs]), mother (MUM) vs community health worker (CHW) clusters.

Figures 2 and 3 provide this information diagrammatically. Figure 2 presents the odds of treatment in MUM vs CHW clusters adjusting for differences between villages, and Figure 3 provides the predicted probability ratios of coverage for each country and shows the higher overall coverage in MUM vs CHW clusters.

## DISCUSSION

Uniformly trained to administer an artesunate suppository in inaccessible villages on the basis of a clinical assessment of danger signs in children, 687 different types of voluntary treatment providers successfully assessed, treated, referred, and followed up treated patients in their communities. The combined data comparing their performance in reaching and treating children in 272 villages provide strong evidence that mothers (MUMs) had a 1.67 higher likelihood of administering prereferral artesunate to children in need of treatment compared with CHWs. This result was hoped for, and underpinned the trial. Despite evidence that mothers could be trained to assess and treat young, sick children in other studies [14, 15], and a proliferation of community programs using CHWs to deliver a wide range of services, no study has convincingly compared performance or treatment access achieved by different types of providers vs each other or vs mothers [32, 33].

A few hours’ delay in malaria can be fatal in a young child, and referral is necessary to complete management and diagnose other conditions. Our earlier trial of rectal artesunate plus referral had extremely high referral completion rates (90%–99%) and very rapid transit to the referral facility [3]. Indeed, the benefit was statistically definite only among patients who needed time to reach a facility because during this period artesunate could halt parasite proliferation and sequestration and confer benefit. Such exceptionally good referral completion rates raised concerns that compliance might not be achievable routinely [34]. Sharing the same concerns, we provided referral notes in this study to motivate patients to complete the referral process, assist facility triage, and expedite curative treatment of children whose symptoms had resolved. Four of 5 patients followed referral advice (81%) in this study, and similar levels of referral completion have now been documented elsewhere [13] and in countries with a high malaria burden [35, 36]. A small proportion of patients defaulted referral, and parents of these children often reported taking no further posttreatment action because the child had improved. Others came too late for treatment to have much chance of providing any material benefit and died en route to hospital [36, 37]. In future work, this finding needs to be emphasized; treatment can only benefit patients if they come early enough for it to be of value.

Prompt diagnosis and treatment is essential to prevent fatal malaria, reduce the numbers of patients who can transmit malaria, and rapidly identify patients with other causes of illness. Among the 4460 patients who declared their inability to control an infection and were treated with prereferral rectal artesunate, a well-tested clinical algorithm was used by treatment providers to verify eligibility for treatment, but parasitology was not undertaken so determination of the pathogen is uncertain. Malaria treatment guidelines specify that an artesunate suppository should be administered to children as soon as a presumptive diagnosis of severe malaria is made because a high risk of death warrants emergency treatment [5]. Prereferral rectal artesunate treatment reduces the risk of death of patients with malaria. However, not all children with danger signs had malaria and the survival of those without malaria, or with incidental malaria and another cause of illness, would depend on speed of diagnosis of alternative infections and management of complications at the facility [38]. In our previous trial, 74% of patients had positive blood slides [3], and a more recent study has shown 82% positivity [35]. As RDTs become available and used routinely, they will reinforce prereferral treatment and reveal the number of patients with danger signs for whom malaria is negative.

Nobody knows how many childhood febrile episodes have danger signs that require prereferral treatment. Prospective, population-based data on prereferral episodes are simply not available, and the regularity with which surveillance is undertaken determines the number of clinical events detected [39]. Monthly surveys—a trade-off between what is practical and reliable—detects fewer clinical events than weekly surveys [40]. The prevalence burden during the period of the study was high. This very large study prospectively following up fevers over 19 months indicates that close to one-third of febrile episodes are apparently accompanied by danger signs. For those who came for treatment, an average of 37% had symptoms of repeated convulsions, altered consciousness, or coma. The study was conducted between 2005 and 2008–2009 when bednet usage was very low and neither ACTs nor RDTs were widely available [41]. National policy changed to ACTs in 2005 in Ghana, in 2006 in Uganda and Tanzania, and in 2007 in Guinea-Bissau. Ghana and Uganda had ACTs for uncomplicated malaria at facilities but not in the community, and first-line antimalarial treatment available in Tanzania and Guinea-Bissau in the study areas was sulfadoxine-pyrimethamine and chloroquine, respectively, throughout the study. The lack of effective treatment and limited bednet usage in the countries undoubtedly contributed to the morbidity reported. Artemisinin combination treatment for malaria has since increased and transmission declines have been reported in many African countries. As evident from our recent study in high malaria prevalence countries, the ideal is that oral artemisinin combination treatment be used in time to prevent uncomplicated malaria episodes from becoming severe [35]. Even in these circumstances there will still be some children who can no longer be treated orally and are in need of prereferral rectal artesunate.

Prereferral rectal artesunate availability through trained treatment providers residing in communities of patients should widen access to diagnosis and accelerate treatment of malaria-positive patients. However, the availability of the intervention for patients distant from facilities did not, on its own, generate adequate demand and use. Given the opportunity for immediate access to a life-saving treatment when a child became acutely ill, only 14% accessed treatment in their communities. Although this low level of coverage could have been caused by insufficient knowledge about the availability and relevance of the intervention, it is also possible that there were too few treatment providers (ie, that they were unavailable when needed) or that morbidity exceeded the ability of few treatment providers per village to cope. Many guardians probably did not go to community-based health providers for a variety of reasons, from anticipated costs to perceptions that the child was not sufficiently ill [42]. Providers were not remunerated, and this might have reduced their availability at their treatment posts as they would be engaged in other income-earning activities. Although incentives might have improved coverage, payment would also have changed the nature of the intervention, irreversibly changed expectations, and prejudiced the approach for scale-up in countries not planning to remunerate community health workers during routine deployment of the intervention.

Knowledge about the importance of this intervention has changed in the past 5 years. Prereferral rectal artesunate and RDTs have been included in community IMCI guidelines [6] and with guidance by WHO and UNICEF, national treatment guidelines have changed to incorporate both; several national government strategies have evolved to incorporate integrated community case management, to screen and treat patients who can safely be managed in the community and refer those who should obtain specialized care [43, 44]. Complementing these efforts, the Global Fund (which finances most malaria control efforts) has begun encouraging country applicants to include community system–strengthening initiatives in proposals wherever relevant to bridge the gap between the formal health system and the community. In consequence, increasing data are becoming available on country upscaling efforts [13, 35, 36]. Skills and competence of the CHWs to manage high-risk patients through training and supervision are being developed. Several African countries now pay community health workers or combine financial and nonfinancial incentives; these approaches are likely to ensure retention and timely availability of trained CHWs for emergency management of malaria using prereferral rectal artesunate.

## Supplementary Data

Supplementary materials are available at http://cid.oxfordjournals.org. Consisting of data provided by the author to benefit the reader, the posted materials are not copyedited and are the sole responsibility of the author, so questions or comments should be addressed to the author.

Supplementary Data

## References

[1] Dondorp A, Nosten F, Stepniewska N, Day NP, White NJ Artesunate versus quinine for treatment of severe falciparum malaria: a randomised trial. Lancet 2005; 366:717–25. 16125588 10.1016/S0140-6736(05)67176-0

[2] Dondorp AM, Fanello CI, Hendriksen ICE et al Artesunate versus quinine in the treatment of severe falciparum malaria in African children (AQUAMAT): an open-label, randomised trial. Lancet 2010; 376:1647–57. 21062666 10.1016/S0140-6736(10)61924-1 PMC3033534

[3] Gomes MF, Faiz MA, Gyapong JO et al Pre-referral rectal artesunate to prevent death and disability in severe malaria: a placebo-controlled trial. Lancet 2009; 373:557–66. 19059639 10.1016/S0140-6736(08)61734-1 PMC2646124

[4] Bahl R, Qazi S, Darmstadt GL, Martines J Why is continuum of care from home to health facilities essential to improve perinatal survival? Semin Perinatol 2010; 34:477–85. 21094421 10.1053/j.semperi.2010.09.001

[5] World Health Organization. WHO guidelines for the treatment of malaria. 3rd ed Geneva, Switzerland: WHO, 2015.

[6] World Health Organization. Integrated management of childhood illness: caring for newborns and children in the community Geneva, Switzerland: WHO, 2011.

[7] Zaman SM, Cox J, Enwere GC, Bottomley C, Greenwood BM, Cutts FT. The effect of distance on observed mortality, childhood pneumonia and vaccine efficacy in rural Gambia. Epidemiol Infect 2014; 142:2491–500. 24565180 10.1017/S0950268814000314 PMC9151291

[8] Bhutta ZA, Chopra M, Axelson H et al Countdown to 2015 decade report (2000–10): taking stock of maternal, newborn, and child survival. Lancet 2010; 375:2032–44. 20569843 10.1016/S0140-6736(10)60678-2

[9] Qazi SA, Stoll BJ Neonatal sepsis: a major global public health challenge. Pediatr Infect Dis J 2009; 28:S1–2. 19106756 10.1097/INF.0b013e31819587a9

[10] Castellani J, Mihaylova B, Evers SMAA et al Out-of-pocket costs and other determinants of access to healthcare for children with febrile illnesses: a case-control study in rural Tanzania. PLoS One 2015; 10:e0122386. 25861012 10.1371/journal.pone.0122386 PMC4393118

[11] Ministry of Health, Adult Morbidity and Mortality Project. The policy implications of adult morbidity and mortality: end of phase 1 report. 1997. Dar es Salaam, Tanzania: United Republic of Tanzania, **1997**.

[12] Carneiro I, Roca-Feltrer A, Griffin JT et al Age-patterns of malaria vary with severity, transmission intensity and seasonality in sub-Saharan Africa: a systematic review and pooled analysis. PLoS One 2010; 5:e8988. 10.1371/journal.pone.0008988 PMC2813874 20126547

[13] Phiri TB, Kaunda-Khangamwa BN, Bauleni A et al Feasibility, acceptability and impact of integrating malaria rapid diagnostic tests and pre-referral rectal artesunate into the integrated community case management programme. A pilot study in Mchinji district, Malawi. Malar J 2016; 15:177. 27000034 10.1186/s12936-016-1237-2 PMC4802711

[14] Kidane G, Morrow R Teaching mothers to provide home treatment of malaria in Tigray, Ethiopia: a randomised trial. Lancet 2000; 356:550–5. 10950232 10.1016/S0140-6736(00)02580-0

[15] Menon A, Joof D, Rowan KM, Greenwood BM Maternal administration of chloroquine: an unexplored aspect of malaria control. J Trop Med Hyg 1988; 91:49–54. 3379654

[16] Sirima SB, Konate A, Tiono AB, Convelbo N, Cousens S, Pagnoni F Early treatment of childhood fevers with pre-packaged antimalarial drugs in the home reduces severe malaria morbidity in Burkina Faso. Trop Med Int Health 2003; 8:133–9. 12581438 10.1046/j.1365-3156.2003.00997.x

[17] Sievers AC, Lewey J, Musafiri P et al Reduced paediatric hospitalizations for malaria and febrile illness patterns following implementation of a community-based malaria control programme in rural Rwanda. World Hosp Health Serv 2008; 44:28–35. 19370834

[18] Bhutta ZA, Lassi ZS Empowering communities for maternal and newborn health. Lancet 2010; 375:1142–4. 20362800 10.1016/S0140-6736(10)60324-8

[19] Bang AT, Bang RA, Tale O et al Reduction in pneumonia mortality and total childhood mortality by means of community-based intervention trial in Gadchiroli, India. Lancet 1990; 336:201–6. 1973770 10.1016/0140-6736(90)91733-q

[20] Bang AT, Bang RA, Sontakke PG Management of childhood pneumonia by traditional birth attendants. Bull World Health Organ 1990; 6:897–905. PMC2486734 7867135

[21] Bang AT, Bang RA, Baitule SB, Reddy MH, Deshmukh MD Effect of home-based neonatal care and management of sepsis on neonatal mortality: field trial in rural India. Lancet 1999; 354:1955–61. 10622298 10.1016/S0140-6736(99)03046-9

[22] Okeke TA, Okafor HU, Uzochukwu B Traditional healers in Nigeria: perception of cause, treatment and referral practices for severe malaria. J Biosoc Sci 2006; 38:491–500. 16762086 10.1017/S002193200502660X

[23] Baskind R, Birbeck G Epilepsy care in Zambia: a study of traditional healers. Epilepsia 2005; 46:1121–6. 16026565 10.1111/j.1528-1167.2005.03505.x PMC1224751

[24] Nations MK, de Sousa MA, Correia LL, da Silva DM Brazilian popular healers as effective promoters of oral rehydration therapy (ORT) and related child survival strategies. Bull Pan Am Health Organ 1988; 22:335–54. 3242735

[25] Pew Forum on Religion and Public Life. Tolerance & tension: Islam and Christianity in sub-Saharan Africa. 2010 Available at: http://www.pewforum.org/2010/04/15/. Accessed 21 September 2016.

[26] Warsame M, Kimbute O, Machinda Z et al Recognition, perceptions and treatment practices for severe malaria in rural Tanzania: implications for accessing rectal artesunate as a pre-referral. PLoS One 2007; 2:e149. 17225854 10.1371/journal.pone.0000149 PMC1764709

[27] Kaona F, Tuba M A qualitative study to identify community structures for management of severe malaria: a basis for introducing rectal artesunate in the under five years children in Nakonde District of Zambia. BMC Public Health 2005; 5:28. 15792501 10.1186/1471-2458-5-28 PMC1079879

[28] Simba DO, Warsame M, Kimbute O et al Factors influencing adherence to referral advice following pre-referral treatment with artesunate suppositories in children in rural Tanzania. Trop Med Int Health 2009; 14:775–83. 19497077 10.1111/j.1365-3156.2009.02299.x

[29] World Health Organization. Pre-referral rectal artesunate treatment of childhood malaria in the community. 2012 Training manual for community health workers Available at: http://www.who.int/tdr/publications/rectal_artesunate/en/ Accessed 21 September 2016.

[30] World Health Organisation. Guidelines for the treatment of malaria 2006.

[31] StataCorp. Stata Statistical Software: release 14. College Station, TX: StataCorp LP, 2009.

[32] Bhattacharyya K, Winch P, LeBan K, Tien M. Community health worker incentives and disincentives: how they affect motivation, retention, and sustainability. Published by the Basic Support for Institutionalizing Child Survival Project (BASICS II) for the United States Agency for International Development. Arlington, Virginia, October 2001.

[33] Bhutta ZA, Lassi ZS, Pariyo GW et al Global experience of community health workers for delivery of health related Millennium Development Goals: a systematic review, country case studies and recommendations for integration into National Health Systems World Health Organization, Geneva, 2010.

[34] von Seidlein L, Deen J Prereferral artesunate in severe malaria. Lancet 2009; 373:522–3. 19059638 10.1016/S0140-6736(08)61735-3

[35] Ajayi IO, Nsungwa-Sabiiti J, Siribié M et al Feasibility of malaria diagnosis and management in Burkina Faso, Nigeria, and Uganda: a community-based observational study. Clin Infect Dis 2016; 63 suppl 5:S245–55. 10.1093/cid/ciw622 PMC5146694 27941101

[36] Siribié M, Ajayi IO, Nsungwa-Sabiiti J, et al. Compliance with referral advice after treatment with pre-referral rectal artesunate: a study in 3 Sub-Saharan African countries. Clin Infect Dis **2016**; 63(suppl 5):S283–9. 10.1093/cid/ciw627 PMC5146699 27941106

[37] Simba DO, Kakoko D, Warsame M et al Understanding caretakers’ dilemma in deciding whether or not to adhere with referral advice after pre-referral treatment with rectal artesunate. Malar J 2010; 9:123. 20459853 10.1186/1475-2875-9-123 PMC2877062

[38] Nadjm B, Amos B, Mtove G et al WHO guidelines for antimicrobial treatment in children admitted to hospital in an area of intense *Plasmodium falciparum* transmission: prospective study. BMJ 2010; 340:c1350. 20354024 10.1136/bmj.c1350 PMC2847687

[39] Snow RW, Craig MH, Newton CRJC, Steketee RW. The public health burden of *Plasmodium falciparum* malaria in Africa: deriving the numbers. Working paper 11. Bethesda, MD: Disease Control Priorities Project, **2003**.

[40] Greenwood BM, Bradley AK, Greenwood AM et al Mortality and morbidity from malaria, among children in a rural area of The Gambia, West Africa. Trans R Soc Trop Med Hyg 1987; 81:478–86. 3318021 10.1016/0035-9203(87)90170-2

[41] World Health Organization. World malaria report 2009. Geneva, Switzerland: WHO, **2009**.

[42] Castellani J, Mihaylova B, Ajayi IO et al Quantifying and valuing community health worker time in improving access to malaria diagnosis and treatment. Clin Infect Dis 2016; 63 suppl 5:S298–305. 10.1093/cid/ciw629 PMC5146701 27941108

[43] United Nations Children's Fund; AMREF; Nefdt R, Ribaira E, Diallo K Scaling up integrated community case management (iCCM) in Eastern and Southern Africa: analysis of programmatic and financial gaps 2012.

[44] World Health Organization. Scaling up community health services to save the lives of children in hard-to-reach areas, 2016 Available at: http://www.who.int/malaria/areas/rapid_access_expansion_2015/race-factsheet-june2016.pdf. Accessed 15 November 2016.

