# Supplementary material for: Pre-referral Rectal Artesunate Treatment by Community-Based Treatment Providers in Ghana, Guinea-Bissau, Tanzania, and Uganda (Study 18): A Cluster-Randomized Trial
Source: Clin Infect Dis. 2016 Dec 6;63(Suppl 5):S312–21. doi: 10.1093/cid/ciw631 (PMC5146703; doi:10.1093/cid/ciw631)
Supplement: Supplementary Data [file supp_63_suppl-5_S312__index.html]

Supplementary Data 

# Pre-referral Rectal Artesunate Treatment by Community-Based Treatment Providers in Ghana, Guinea-Bissau, Tanzania, and Uganda (Study 18): A Cluster-Randomized Trial

## Supplementary Data

Supplementary Data

- Supplementary Data - Pdf file
