# Supplementary material for: Pre-referral Rectal Artesunate Treatment by Community-Based Treatment Providers in Ghana, Guinea-Bissau, Tanzania, and Uganda (Study 18): A Cluster-Randomized Trial
Source: Clin Infect Dis. 2016 Dec 6;63(Suppl 5):S312–21. doi: 10.1093/cid/ciw631 (PMC5146703; doi:10.1093/cid/ciw631)
Supplement: Supplementary Data [file supp_ciw631_ciw631supp.pdf]

Supplementary Table 1: Coverage: Crude estimates, weighted by reported pre-referral episodes per village

	Number of villages	Total NPOs	Total treated	Crude Proportion treated	Median coverage	(IQR)	Mean coverage	SD (mean)
**Guinéa-Bissau**								
MUM	23	1508	176	11.7	7.9	0 – 25.3	18.9	26.5
CHW	26	2315	105	4.5	4.7	0 – 36.4	20.9	31.5
TH	25	1614	152	9.4	13.3	2.2 – 23.2	18.6	21.7
**Ghana**								
MUM	24	943	384	40.7	40.4	21.3 – 67.7	43.2	26.8
CHW	24	878	186	21.1	21.4	7.5 – 40.8	30	28.4
COM	26	1145	360	31.4	28.8	11.4 – 44.4	30.1	22.8
**Tanzania**								
MUM	34	9675	1399	14.5	17	7.5 – 23.7	18.5	15.3
CHW	33	8114	670	8.2	5.9	4.0 – 11.4	10.1	11.9
**Uganda**								
MUM	31	2576	486	18.9	28.1	9.2 – 51.5	32.7	28.9
CHW	26	3049	503	16.5	18.1	5.8 – 35.1	26.3	24.1
